# Disruption of Intracellular Calcium Homeostasis Leads to ERLIN2-Linked Hereditary Spastic Paraplegia in Patient-Derived Stem Cell Models

**DOI:** 10.1155/2023/4834423

**Published:** 2023-06-16

**Authors:** Xintong Zhu, Xiaoyin Tan, Junwen Wang, Limeng Dai, Jia Li, Xingying Guan, Ziyi Wang, Mao Zhang, Jun Hu, Yun Bai, Hong Guo

**Affiliations:** ^1^Department of Medical Genetics, College of Basic Medical Sciences, Army Medical University, Chongqing 400038, China; ^2^NHC Key Laboratory of Birth Defects and Reproductive Health (Chongqing Key Laboratory of Birth Defects and Reproductive Health, Chongqing Population and Family Planning Science and Technology Research Institute), Chongqing 400020, China; ^3^Department of Neurology, Southwest Hospital, Army Medical University, Chongqing 400038, China; ^4^Department of Gynaecology and Obstetrics, Xinqiao Hospital, Army Medical University, Chongqing 400037, China

## Abstract

Hereditary spastic paraplegia (HSP) is a category of neurodegenerative illnesses with significant clinical and genetic heterogeneity. Homozygous truncated variants of the *ERLIN2* gene lead to HSP18 (MIM #611225). However, it is still unclear whether there is an autosomal dominant pathogenic pattern. The specific molecular mechanism needs to be investigated. We generated patient-derived iPSC models to study the mechanism of *ERLIN2* heterogeneous variants leading to HSP. We identified a heterozygous missense variant p.Val71Ala of *ERLIN2* in an HSP family. Based on IP-mass spectrometry, we found that the ERLIN2 heterozygous missense variant protein recruited the ubiquitin E3 ligase RNF213 to degrade IP3R1. The degradation of IP3R1 leads to the reduction of intracellular free calcium, which triggered endoplasmic reticulum (ER) stress-mediated apoptosis. Calcium homeostasis imbalance inhibited the MAPK signaling pathway that contributed to decreased cell proliferation. In summary, these results suggest that the autosomal dominant inheritance of heterozygous missense variants in *ERLIN2* is a novel pathogenic mode of HSP. Furthermore, the disruption of intracellular calcium homeostasis is the pathological mechanism.

## 1. Introduction

Hereditary spastic paraplegia (HSP) is a category of neurodegenerative diseases with a wide range of clinical and genetic characteristics. The common clinical symptoms included increased muscle tension in the lower limbs, active and hyperactive tendon reflexes, positive pathological reflex, and scissor gait [1]. Slowly developing muscular weakening and sluggish spastic paraplegia of the lower limbs define the most specific clinical manifestations of this disease [2]. HSP causes axonal degeneration and demyelination of upper motor neurons, with the lengthy fiber bundles in the spinal cord affected. More than 80 HSP pathogenic gene loci have been discovered according to the OMIM database, with more than 60 pathogenic genes cloned [3]. The known HSP genes take part in a range of cellular activities such as lipid metabolism, vesicle transport, cell signaling, and intracellular calcium homeostasis. Calcium homeostasis has attracted increasing attention in the study of motor neuron disease (MND) [4–6].

Intracellular calcium levels in neurons are particularly sensitive, and it plays a role in neuronal excitability, synaptic signaling, neurotransmitter release, activation of specific calcium-related signaling pathways, and gene transcription [7]. Intracellular calcium homeostasis is principally regulated by the cell membrane, endoplasmic reticulum (ER), and mitochondria-resident Ca^2+^ transporters. IP3Rs are tetrameric complexes found in the ER membrane triggered by IP3 produced from plasma membrane G-protein-coupled receptors, resulting in Ca^2+^ efflux from the ER [8]. Therefore, the IP3/IP3Rs pathway maintains intracellular calcium levels under the action of the ER-associated degradation pathway (ERAD). Calcium homeostasis imbalance leads to nerve cell activity disturbance, not only affecting the normal physiological activities of neurons but also diminishing the structural integrity and vitality [9–11]. Here, the study mainly explored the effect of ERLIN2 heterozygous missense variants on HSP.

ER lipid raft-associated protein 2 is encoded by the human *ERLIN2* gene, which is located on chromosome 8p11.23. The ERLIN2 protein consists of the SPFH domain, oligomerization domain, and hydrophobic domain. The SPFH domain is the primary functional region, which is found in the ER's transmembrane area and forms a heterooligomeric complex with ERLIN1 to act in the ERAD pathway [12]. Since ERLIN2 is involved in the degradation of IP3R1 in the ERAD pathway, we speculate that it may play an important role in the regulation of intracellular calcium homeostasis. HSP has previously been shown to link to the biallelic variants in *ERLIN2* (MIM #611225) [13], but it is still uncertain whether heterozygous missense mutations cause HSP, and the pathological mechanism needs to be investigated. Therefore, we constructed a patient-derived iPS cell model to explore its molecular mechanism.

In this study, we found that a novel *ERLIN2* variant was linked to autosomal dominant HSP in a Chinese family. The *ERLIN2* p.Val71Ala variant was discovered to cause HSP by affecting intracellular calcium homeostasis, which might cause ER stress in patient-derived iPSC-induced neural stem cells (NSCs). Our results highlight intracellular calcium homeostasis mediated by inositol 1,4,5-triphosphate signal transduction as a candidate pathway for the development of future therapeutic interventions. Based on patient-derived iPSC-induced NSCs, we further explained that HSP caused by the *ERLIN2* p.Val71Ala mutant is a neurodevelopmental disorder.

## 2. Materials and Methods

### 2.1. Patients

**Figure 1:**
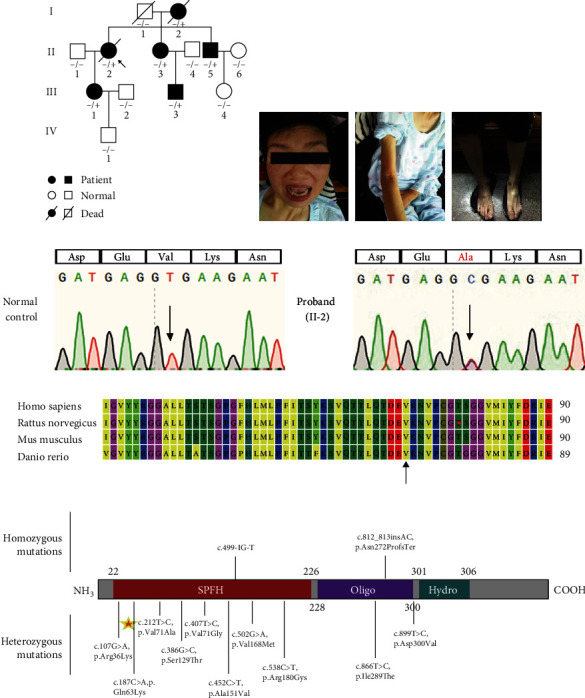
Identification of ERLIN2 heterozygous missense variants in the hereditary spastic paraplegia (HSP) family and molecular characterization. (a) Pedigrees of HSP families. Shaded symbols are affected with features of HSP, white symbols are normal, and a plus sign and a minus sign mean the heterozygous variant of ERLIN2 missense variant c.212T>C: p.Val71Ala. (b) Clinical features of individual II-2 and II-5, such as amyostasia, amyotrophy, and myospasm. (c) The partial nucleotide sequences of exon 4 of *ERLIN2* show the c.212T>C: p.Val71Ala variant in the affected family members (II-2, II-3, II-5, III-1, and III-3) and the normal control. (d) Multiple sequence alignment of the *ERLIN2* partial region. The arrow indicates the amino acid position 71 in human *ERLIN2*. (e) The ERLIN2 protein consists of three main domains, the SPFH domain (residues 22–226), the oligomerization domain (residues 228–300), and a short hydrophobic patch (residue at position 305). The c.212T>C: p.V71A variant is located in the SPFH domain, and the asterisk indicates the variant originated from our patient.

This study included five members of a three-generation family (Figure 1(a)). We used common clinical standards to clinically evaluate patients and conducted electromyography (EMG), electroencephalogram (EEG), and other examinations. We took peripheral blood from these five patients for nucleic acid extraction and cell reprogramming. We obtained written informed consent from all of the study participants. All research was conducted according to relevant guidelines and regulations. Cells used for reprogramming were derived from patients I-1 (iPS-GDL), II-5 (iPS-GJH), and III-1 (iPS-YCY). Our study was approved by the Ethics Committee of the Army Medical University, ethics approval number 2022-10-01.

### 2.2. Variant Identification

The unaffected family member (I-1) and the family members with autosomal dominant HSP were subjected to whole-exome sequencing (II-3, II-5). For the three family members, the total number of identified variants was 63,760. Using the databases 1000 genome, dbSNP, ClinVar, and Exome Aggregation Consortium, we eliminated variations in synonymous variants and variants with allele frequencies greater than 0.01 in order to discover harmful variants (ExAC). Only one variant fits the autosomal dominant inheritance pattern. The variant prediction tools SIFT, FATHMM, MutationTaster, and PROVEAN were used to predict the harmfulness of mutations. Finally, we identified the c.212T>C variant in ERLIN2.

### 2.3. Cell Reprogramming and iPSC Culture

Informed consent was obtained from all patients before taking peripheral blood samples. The protocol we used for reprogramming was described by Stemcell (Stemcell, 05924). The hiPSCs were cultured in PGM1 human iPSC medium (Cellapy, CA1007500) on Matrigel-coated (Corning, 354277) 6-well plates at 37°C with 5% CO_2_. The medium was changed every day until the hiPSC confluence reached 80-90%. HiPSCs were passaged every 3-4 days by using a 0.5 mM EDTA-PBS solution (Cellapy, CA3001500). We reprogrammed two patient-derived hiPS cell lines as p.Val71Ala groups: iPS-GJH and iPS-YCY, with the I-5 (iPSC-GDL) as a normal control within the family. All cell lines were between 20 and 30 passages before reprogramming.

### 2.4. Differentiation of MNs from iPSCs

Motor neuron differentiation followed previously published protocols. iPSC monolayers were induced to NPCs under serum-free conditions, by dual SMAD inhibition using dorsomorphin and SB431542 (Stemcell, 72234), coupled with GSK-3 inhibition by CHIR-99021 (Stemcell, 72052) and *γ*-secretase inhibition by compound-E (Stemcell, 73954). Following neutralization, soluble RA (Stemcell, 72262), SHH (Beyotime, P7212), and purmorphamine (Stemcell, 72204) were used to specify spinal MN fate. Finally, BDNF (Biow, BK0184), GDNF (Beyotime, P7514), NT-3 (NovoProtein, C079), and CNTF (NovoProtein, C098) were added to promote the maturation of motor neurons.

### 2.5. Measurement of Calcium Imaging

Cells were loaded with Fluo-4 AM (Beyotime, 2 *μ*M) for 30 min at 37°C in NSA medium. The cells were then washed three times with D-PBS and cultured in NSA medium. The Petri dishes were placed on confocal microscope platform. When the excitation wavelength was 488 nm, the change was monitored in fluorescence intensity of single cells. Next, 1 *μ*M TG was carefully introduced into the culture dish at the 100th second to trigger calcium release from the ER, and a total of 1300 seconds were recorded. The WT group selected 45 cells to record, and the p.Val71Ala group selected iPS-GJH cell line-derived neural stem cells to record 45 cells, *N* = 3 experiments. *F*0 was the background fluorescence value of untreated cells, and *F* was the real fluorescence value of cells. Δ*F* = *F* − *F*0, and Δ*F*/*F*0 represented the change of cell fluorescence intensity.

### 2.6. Flow Cytometry Analysis Experiments

Annexin V-FITC/PI apoptosis detection kits (Beyotime, C1062M) were used to detect apoptosis. About 1 million cells were resuspended with cold D-PBS and bound buffer supplemented with Annexin V-FITC and PI at 37°C for 10 min. Finally, BD Accuri C6 plus flow cytometer was used to analyze the cells.

A cell cycle detection kit (Beyotime, C1052) was used in the cell cycle. Briefly, cells were dissociated into single cells by Accutase (Sigma Technology, USA, A6964), and about 2 million cells were fixed in 75% ethanol at 4°C overnight. After washing the cells with cold D-PBS three times, we added propidium iodide (PI) into cell suspension, and the cells were incubated at room temperature for another 30 min in the dark. Finally, BD Accuri C6 plus flow cytometer was used to analyze the cells. Then, the cells were divided into each cell cycle phase based on the PI intensity in FlowJo 10.5.0, and the proportion of each phase was calculated.

### 2.7. Western Blot

For protein extraction, neural stem cells were harvested and lysed in Pierce™ RIPA buffer (Thermo, 78510) (100X Phosphatase Inhibitor Cocktail, GRF101.100X Protease Inhibitor Cocktail, GRF102). The lysates were quantified by the Enhanced BCA Protein Assay Kit (Beyotime, P0010), and equal amounts of proteins were loaded for western blot (WB) assay. Antibodies used for WB were anti-ERLIN2 (Cell Signaling Technology, 2959, 1 : 1000), anti-IP3R1 (Cell Signaling Technology, 8568, 1 : 1000), anti-RNF213 (Santa Cruz Biotechnology, sc-293391, 1 : 500), anti-caspase 3 (Abclonal, A16793), anti-cleaved caspase 3 (Beyotime, AC033), anti-CHOP (Cell Signaling Technology, 2895, 1 : 1000), anti-Erk1/2 (Cell Signaling Technology, 9102S, 1 : 1000), anti-phospho-Erk1/2 (Thr202/Tyr204) (Cell Signaling Technology, 4370T, 1 : 1000), and anti-P53 (Abcam, ab26, 1 : 1000). Briefly, the proteins were separated by 12% SDS-PAGE (Bio-Rad, TGX Stain-Free FastCast Acrylamide Kit 12% Cat#1610185) and transferred to a PVDF membrane. After being blocked with QuickBlock™ Western (Beyotime, P0252) for 1 h at room temperature, the membrane was incubated overnight at 4°C with the primary antibodies. Then, the membranes were incubated with HRP-conjugated goat anti-rabbit IgG (GtxRb-003-DHRPX, ImmunoReagents, 1 : 10000) for 2 h at room temperature. WB experiments were repeated at least three times. The quantification of WB bands was performed using ImageJ software (https://imagej.nih.gov/ij/docs/guide/146-30.html).

### 2.8. Quantitative RT-PCR

Total RNA was isolated using an RNeasy mini kit (QIAGEN) with DNase I treatment, and cDNA was prepared using a PrimeScript™ RT reagent kit with gDNA Eraser (Perfect Real Time) (TaKaRa Bio, RR047A). Quantitative RT-PCR was performed using TB Green Premix Taq™ II (TIi RNaseH Plus) (TaKaRa Bio, RR820A) on an SYBR Real-Time PCR System (Bio-Rad). The details of the qRT-PCR primers are described in Supplementary Table 1.

### 2.9. Statistical Analysis

Statistical analysis was performed with SPSS 26.0 (SPSS Statistic IBM). Data are represented as mean ± SD, with 95% confidence intervals. A two-tail unpaired Student's *t*-test (data with normal distribution) was conducted to compare two independent groups. We used a paired *t*-test (normal distribution) to compare dependent measurements. A critical value for significance of *p* < 0.05 was used throughout the study. ^∗^*p* < 0.05, ^∗∗^*p* < 0.01, and ^∗∗∗^*p* < 0.001.

## 3. Results

### 3.1. A Novel Heterozygous Missense Variant in *ERLIN2* Was Identified in an HSP Family

Late-onset HSP was worsened by axonal peripheral neuropathy and cognitive impairment in patients from an autosomal dominant family (Figure 1(a)). The proband (II-2) was a 58-year-old woman who had been suffering from growing muscle atrophy and weakness. The patient began having gait disturbances in her 30s and then vision problems in her 50s, followed by slurred speech, difficulties in eating, and limb weakness by the time she was 58, when a wheelchair was needed, and she died at the age of 60 due to respiratory muscle weakness. Furthermore, as the condition progressed, patients began to experience cognitive impairment. The patient's SPRS score was 44. The results of the auxiliary examination showed that the proband's brain MRI showed no obvious abnormality, while the spinal MRI suggested cervical spine degeneration, and electromyography suggested neurogenic damage. II-5, a 35-year-old male, had adolescent onset, beginning with lower limb muscle weakness, which progressed to lower extremity spasm and then suffered from increased tension and muscle atrophy, accompanied by muscle fibrillation, and in the later stage of the disease, the patient was unable to walk, but the patient's upper limbs, head, and trunk were unaffected; the SPRS score was 39; II-3, III-1, and III-3 just could not walk steadily (Table 1, Figure 1(b), and Supplementary Table S1). The phenotypes in affected family members conform to HSP with a varied onset age.

To clarify the genetic etiology of this HSP pedigree, we first performed fragment analysis of neuromuscular disease in the proband and found no dynamic variants (such as *SCAs* and *C9orf72*). Then, we used whole-exome sequencing (WES) to find the causal variant (details on variant screening and identification can be found in Variant Identification). Combining WES with genetic and bioinformatic analysis of the sequencing results determined that the variant was c. 212T>C (p.Val71Ala) at exon 4 of the *ERLIN2* gene (NM 007175.6). The results of Sanger sequencing on both unaffected and affected members of the family revealed the variant cosegregation (Figure 1(c) and Supplementary Figure S1). The prediction programs gave p.Val71Ala a high pathogenic effect score (Table 1). Furthermore, the p.Val71Ala variant occurs in a highly conserved area in other species (Figure 1(d)). This variant is in the SPFH domain (Figure 1(e)), which is the key location of protein interaction. Earlier studies have found that variants in the SPFH domain of the *ERLIN2* gene could cause HSP or ALS [14–16], but the heterozygous pathogenic mode is not clear, and the molecular mechanism remains to be elucidated. Patients with the p.Val151Ala variant of *ERLIN2* had lower extremity muscle atrophy, increased muscle tone, pathological reflexes and hyperreflexia, and other motor system disorders, as well as obvious paresthesias and seizures. The patients with the p.Ser129Thr, p.Gln63Lys, p.Asp69Val, and p.Val168Met variants of *ERLIN2* had the same phenotypes as our patients (Table 2). In general, we elicited that *ERLIN2* can cause hereditary spastic paraplegia through autosomal dominant inheritance.

### 3.2. Differentiation of Patient-Derived iPSC into Motor Neurons Was Affected in the Presence of *ERLIN2* p.Val71Ala Variant

**Figure 2:**
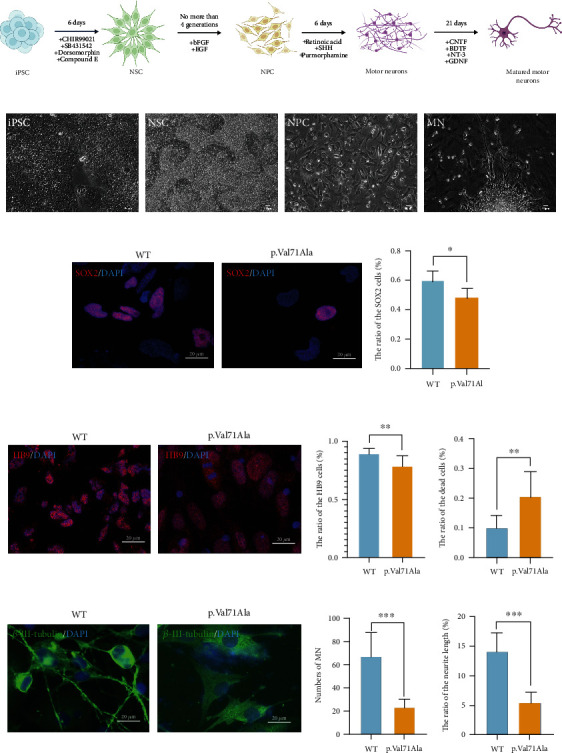
The differentiation from iPSC into motor neurons. (a) Schematic diagram of the process of motor neuron differentiation. iPSC = induced pluripotent stem cells; NSCs = neural stem cells; NPCs = neural progenitor cells; MN = motor neurons. (b) Various cell morphologies at the differentiation stage of motor neurons. (c) iPS-GDL as the WT, iPS-GJH as the p.Val71Ala, iPSC-induced NSCs were immunofluorescence stained with SOX2, and the number of SOX2-positive cells was calculated (mean ± SD, *n* = 10 images, *N* = 3 experiments, ^∗∗∗^*p* < 0.001). (d) iPSC-induced NPCs were immunofluorescence stained with HB9. The number of HB9-positive cells and dead cells was calculated (mean ± SD, *n* = 10 images, *N* = 3 experiments, ^∗∗^*p* < 0.01). (e) Immunofluorescence staining of motor neurons on day 21 of maturation showed that the number of MNs was smaller and the axon length of motor neurons was significantly shorter in the p.Val71Ala group than in the WT group. The green was *β*-III-tubulin, and the blue was DAPI (mean ± SD, *n* = 10 images, *N* = 3 experiments, ^∗∗∗^*p* < 0.001).

To study the pathogenesis of HSP caused by the *ERLIN2* p.Val71Ala variant, we used reprogramming techniques to edit patients (iPS-GJH and iPS-YCY) and normal controls (iPSC-GDL) in the family line, and after karyotype analysis and pluripotency verification, we obtained mature iPS cell lines (Supplementary Figure S2). Based on the patient-derived iPSC model, we adopted a protocol of dual SMAD inhibition to induce the differentiation of motor neurons (Figure 2(a)), resulting in the *in vitro* differentiation of motor neurons that were successfully obtained and enriched (Figure 2(b)). We used iPS cell lines from individuals who exhibited no clinical phenotype in the lineage and do not carry the *ERLIN2* variant as controls.

By evaluation, we found that the differentiation efficiency of patient-derived IPS was lower than that of the WT within the family, resulting in only a limited number of motor neurons at the end. The proportion of NSCs in the p.Val71Ala group was considerably lower than that in the WT group at the NSC stage (Figure 2(c)). After developing into neural progenitor cells (NPCs), the percentage of NPCs in the p.Val71Ala group was lower and the dead cell percentage was higher (Figure 2(d)). At the same time, we also found that the number of MNs was smaller and the length of MN axons was significantly shorter in the p.Val71Ala group than in the WT by measuring the axon length of motor neurons at day 21 of maturation (Figure 2(e)), quantification indicating the ratio of the position of the neurite terminal from the nucleus center to the diameter of the nucleus, *p* < 0.001. Thus, the variant dramatically reduces the efficiency of differentiation from patient-derived iPSC into motor neurons and inhibits the ability to differentiate into motor neurons.

### 3.3. The p.Val71Ala Variant in *ERLIN2* Led to the Degradation of IP3R1 and Disturbance of Calcium Homeostasis by Recruiting RNF213

**Figure 3:**
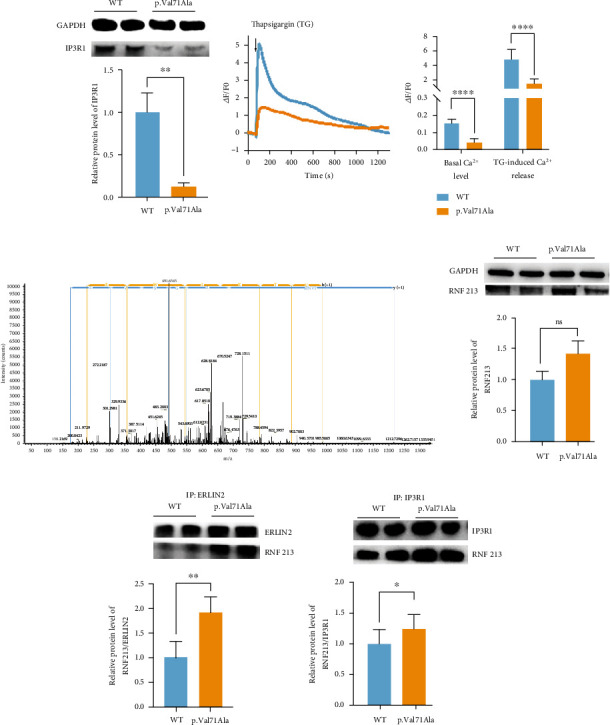
The p.V71A variant in *ERLIN2* resulted in the degradation of IP3R1 and the disturbance of calcium homeostasis by recruiting RNF213. (a) The IP3R1 protein was significantly downregulated in *ERLIN2* p.V71A-mutant NSCs (mean ± SD, *N* = 3 experiments, ^∗∗∗^*p* < 0.001). (b) The Fluo-4 AM fluorescent probe labeled intracellular calcium ions, and the calcium imaging was scanned by confocal microscopy. Thapsigargin (TG) was added at the time of 100 s. The intracellular basal calcium concentration and calcium release after TG stimulation were analyzed (mean ± SD, *n* = 45 cells, *N* = 3 experiments, ^∗∗∗∗^*p* < 0.0001). (c) Anti-ERLIN2 immunoprecipitates (IP) from WT and p.Val71Ala NSCs (iPS-GJH). The mass spectrometry showed that compared with the WT, the p.Val71Ala interacted with RNF213. (d, e) In NSCs, the expression of RNF213 was not significantly different between the WT and p.Val71Ala. The p.Val71Ala ERLIN2 interaction with RNF213 was indeed significantly increased compared to the WT (mean ± SD, *N* = 3 experiments, ^∗^*p* < 0.05). (f) In the p.Val71Ala group, the interaction between IP3R1 and RNF213 was significantly increased (mean ± SD, N = 3 experiments, ^∗∗∗^*p* < 0.001), with iPS-GDL as the WT and iPS-GJH as the p.Val71Ala.

Previous studies have indicated that the ERLIN2 protein plays a role in the ER-associated degradation (ERAD) pathway to degrade activated IP3R1 (which is mainly expressed in neurons) via the ubiquitin-proteasome pathway (UPP) to maintain intracellular calcium homeostasis [17]. In the presence of the *ERLIN2* p.Val71Ala variant, the IP3R1 level was dramatically reduced in neural stem cells (Figure 3(a)). We used Fluo-4 fluorescent probe to label intracellular calcium ions, and the cells were scanned for calcium imaging by confocal microscopy (Figure 3(b)). When the cell fluorescence was stable, 1 *μ*M thapsigargin (TG) was added to the cells to induce calcium release from the ER. We discovered that the p.Val71Ala group's basal intracellular calcium ion level was lower than the WT group's and that calcium ion release in the p.Val71Ala group was prevented following induction with TG.

To explore whether the *ERLIN2* p.Val71Ala mutant recruited a new ubiquitin ligase to degrade IP3R1 and reduce its expression level, we performed immunoprecipitation (IP) of total proteins in NSCs from WT and p.Val71Ala with anti-ERLIN2 antibody and analyzed the products by mass spectrometry. RNF213 was identified, which interacts with the ERLIN2 protein in the p.Val71Ala group compared to the WT group (Figure 3(c)). RNF213 is an E3 ubiquitin-protein ligase that is involved in the noncanonical Wnt signaling pathway in vascular development [18]. By using western blot, we first verified that the ERLIN2 mRNA and protein levels were not significantly altered (data not shown), and then, we discovered that the overall RNF213 protein in the p.Val71Ala group did not change considerably (Figure 3(d)), but the protein interacting with ERLIN2 did (Figure 3(e)). Furthermore, the IP-WB data revealed a considerable increase in IP3R1 interaction with RNF213 (Figure 3(f)). As a result, we hypothesize that the *ERLIN2* p.V71A variation causes pathological ER stress in NSCs by increasing IP3R1 degradation after recruiting RNF213, which alters calcium efflux in the ER lumen and disrupts intracellular calcium homeostasis, thus leading to apoptosis.

### 3.4. *ERLIN2* p.Val71Ala Variant Increased NSC Apoptosis via ER Stress Which Can Be Rescued by TUDCA

**Figure 4:**
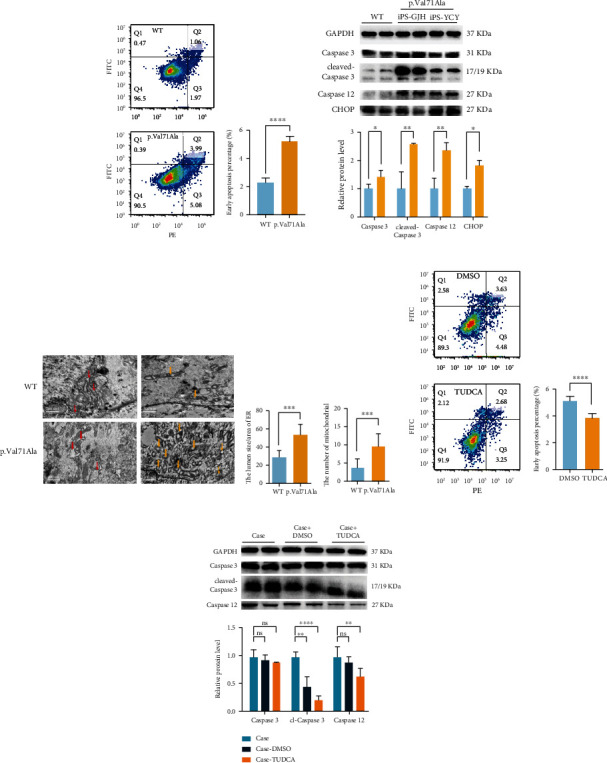
The *ERLIN2* p.V71A mutant increased the apoptosis of NSCs via ER stress and can be rescued by TUDCA. (a) Apoptosis was detected by flow cytometry after staining with Annexin V-FITC apoptosis detection kit. The early apoptosis percentage was expressed as Q3. The bar chart represents the quantification of the result (mean ± SD, *N* = 3 experiments, ^∗∗∗∗^*p* < 0.0001) with iPS-GDL as the WT and iPS-GJH as the p.Val71Ala. (b) Western blot showed that caspase 3, cleaved-caspase 3, caspase 12, and CHOP were significantly upregulated (mean ± SD, *N* = 3 experiments, ^∗^*p* < 0.05, ^∗∗^*p* < 0.01). (c) The representative EM images and quantification of mitochondria number and the ER lumen/area in WT and p.Val71Ala NSCs (mean ± SD, *n* = 25 images, *N* = 3 samples, ^∗∗∗^*p* < 0.01, ^∗∗∗∗^*p* < 0.001), iPS-GDL as the WT group and iPS-GJH as the p.Val71Ala group. The red arrows represent the ER, and the yellow arrows represent the mitochondria. (d) The iPS-GJH-derived NSCs were treated with 100 *μ*M DMSO and 100 *μ*M tauroursodeoxycholate (TUDCA). Apoptosis was detected by flow cytometry after staining with Annexin V-FITC apoptosis detection kit. The early apoptosis percentage was expressed as Q3. The bar chart represents the quantification of the result (mean ± SD, *N* = 3 experiments, ^∗∗∗∗^*p* < 0.0001). (e) Western blot showed that the protein level of caspase 3 was not significantly different, while the cleaved-caspase 3 and caspase 12 had been significantly downregulated when the cells were treated with TUDCA (mean ± SD, *N* = 3 experiments, ^∗^*p* < 0.05, ^∗∗^*p* < 0.01, ^∗∗∗^*p* < 0.001).

We used flow cytometry to analyze the apoptotic level of cells to investigate whether the variant caused an increase in apoptosis. The number of apoptotic cells was significantly higher in the p.Val71Ala group than in the WT group (Figure 4(a)). Caspase 3, cleaved-caspase 3, and caspase 12 are apoptosis-related proteins that indicate the amount of apoptosis and play a vital role in apoptosis. According to western blot, the expression of caspase 3 and caspase 12 was higher in the p.Val71Ala group than in the WT group, and the level of cleaved-caspase 3 protein was 4.11 times higher than that in the WT (Figure 4(b)). In conclusion, the level of apoptosis was much higher in the p.Val71Ala than in the WT group.

As previously stated, in the presence of the *ERLIN2* p.Val71Ala variant, the protein IP3R1 was heavily degraded, reducing its expression and affecting calcium efflux from the ER. It potentially causes ER stress due to calcium overload in the ER lumen. As a result, we detected the ER stress-related protein CHOP, a transcription factor that regulates the expression of apoptosis-related proteins like caspase 3 and caspase 12, and discovered that its expression was significantly increased in the p.Val71Ala group (Figure 4(b)), indicating that the level of ER stress in the *ERLIN2* p.Val71Ala mutant neural stem cells is significantly increased. Using RT-PCR, we detected the gene expression of ubiquitin protease response- (UPR-) related genes (*GRP78*, *PERK*, *XBP1*, *ATF6*, *ATF4*, *EIF2S1*, and *IRE1*) and found no significant increase, even though there was a drop in expression (Supplementary Figure S3A). We used electron microscopy (EM) to measure the distance between the proximal and distal sides of the ER and discovered that the ER was significantly dilated in the p.Val71Ala group, as well as a significant increase in the number of mitochondria (Figure 4(c)). As previously reported, decreased intracellular calcium concentration leads to increased mitochondrial cleavage. Therefore, the *ERLIN2* p.Val71Ala mutant causes greater ER stress in the NSCs, which leads to increased apoptosis.

Tauroursodeoxycholate (TUDCA) is an ER stress inhibitor that inhibits the expression of apoptosis-related proteins such as caspase 3 and caspase 12. We used 100 *μ*M TUDCA and the same volume of DMSO to treat patient-derived iPSC-induced NSCs. Flow cytometry was used to measure apoptosis levels after 48 hours of therapy, and TUDCA showed a significant improvement in apoptosis (Figure 4(d)), while the same was true for the WT group (Supplementary Figure S3B). Following that, we searched for apoptotic molecules in the TUDCA treatment group, and the western blot revealed that caspase 3 expression was not lowered, but cleaved-caspase 3 and caspase 12 expressions were considerably inhibited (Figure 4(e)). TUDCA, an ER stress inhibitor, was found to be effective in inhibiting the *ERLIN2* p.V71A mutant's increased apoptosis.

### 3.5. *ERLIN2* p.Val71Ala Variant Decreased NSC Proliferation by the MAPK Signaling Pathway

**Figure 5:**
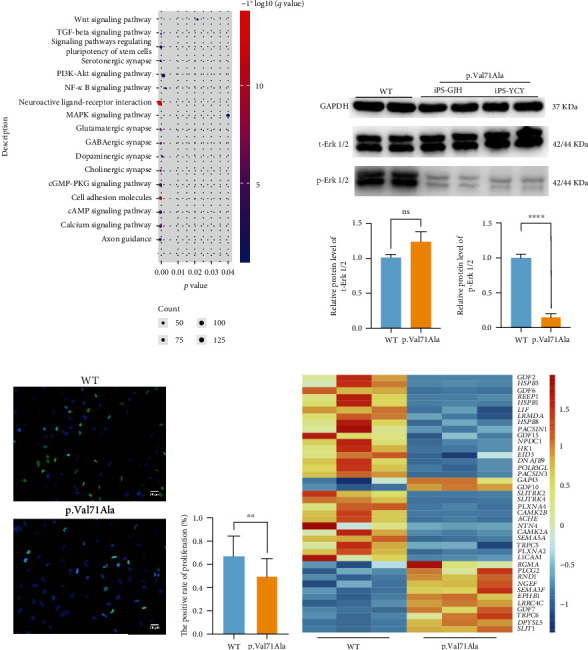
Impaired proliferation and abnormal differentiation of ERLIN2 p.V71A-mutant NSCs. (a) The dot plot showed the significantly enriched categories of KEGG of the differentially expressed genes in the p.Val71Ala based on the WT. (b) The western blot showed that the protein expression levels of t-Erk1/2 and p-Erk1/2 in the MAPK signaling pathway were significantly downregulated (mean ± SD, *N* = 3 experiments, ^∗∗∗^*p* < 0.001). (c) Cell proliferation was detected using the BeyoClick™ EDU-488 kit. The green fluorescence indicates cells that have the ability to proliferate, the blue fluorescence indicates the nucleus, and the bar chart shows the percentage of proliferated cells (mean ± SD, *n* = 15 images, ^∗∗^*p* < 0.01). (d) The heat map demonstrates differentially expressed genes related to motor neuron differentiation in the p.Val71Ala group compared to the WT group, iPS-GDL as the WT, and iPS-GJH and iPS-YCY as the p.Val71Ala.

To investigate how the *ERLIN2* p.Val71Ala mutant affects proliferation and differentiation, we performed transcriptome sequencing on iPSC-induced NSCs. We recruited the differential expressions of several genes related to cell proliferation and differentiation signaling pathways (Figure 5(a)), such as the NF-*κ*B signaling pathway (which is regulated by intracellular calcium concentration and CCaMK), the MAPK signaling pathway, the TGF signaling pathway, and other signaling pathways that regulate motor neuron differentiation. The transcription level of genes related to the MAPK signaling pathway, which is directly regulated by Calcium (Ca^2+^)/calmodulin (CaM)-dependent protein kinase (CCaMK), was downregulated. Western blot analysis showed that the phosphorylation level of the ERK protein was significantly reduced (Figure 5(b)). We then examined the proliferation ability of NSCs using the EdU Cell Proliferation Kit and discovered that the proliferation of NSCs was greatly reduced in the p.Val71Ala group (Figure 5(c)).

In addition, by analyzing differentially expressed genes, we revealed the downregulated expression of genes that promote cell differentiation into motor neurons (*CAMK2B* and *L1CAM*) and axon genesis and elongation (*NTN4*, *TRPC5*, *PLXNA4*, and *SEMA5A*) (Figure 5(d)). RT-PCR was performed for validation (Supplementary Figure S4). These findings provide an explanation for the low number of patient-derived iPSC-induced motor neurons and axon dysplasia that we previously discovered.

## 4. Discussion

Hereditary spastic paraplegias (HSPs) are a set of neurodegenerative disorders defined by the degradation of long axons inside the corticospinal tract, resulting in increasing lower limb stiffness and weakening [19, 20]. The clinical characteristics are substantially overlapping with amyotrophic lateral sclerosis (ALS), Charcot-Marie-Tooth disease (CMT), spinocerebellar ataxias (SCA) [21–23], and other motor neuron diseases, causing problems with clinical diagnosis. By means of whole-exome sequencing and bioinformatic analysis [24], as well as RNAseq analysis of patient-derived iPSC-derived NSCs [25], we did not find variants in the coding regions of genes known to be involved in motor neuron disease or differences in their expression levels. The pathogenic variant in this family was therefore identified as the ERLIN2 heterozygous missense variant p.Val71Ala.

Various cellular activities associated with the ER have been shown to play an important role in HSP, such as nonfolding protein response, lipid metabolism, substance transport, and regulation of intracellular calcium homeostasis. *ERLIN2* biallelic variation has previously been shown to play a role in HSP type 18 (HSP 18) [26]. Patients are generally presented with muscle atrophy, upper and lower limb spasticity, and hyperreflexia, and a few patients are also accompanied by intellectual disability. However, the heterozygous pathogenic pattern of the ERLIN2 gene has not been confirmed, and only a few cases have reported similar phenotypes (Table 2) [14, 15, 27]. In this study, a heterozygous variant of ERLIN2 was also found in a family with autosomal dominant inheritance, and its pathogenic mechanism was studied by molecular biology technology.

Calcium homeostasis is a common and an essential mechanism of HSP, involving ER-related proteins such as ERLIN2, ERLIN1, IP3R1, and RNF170, which interact with ER lipid rafts. Variants in them may bring changes in lipid raft signaling, leading to motor neuron diseases such as amyotrophic lateral sclerosis (PLS), HSP, and SCA (Table 3) [28]. As an important calcium receptor, the turnover of IP3R1 was finely regulated. Although variants in different genes lead to different clinical features of the disease, we found that biallelic variants in *ERLIN2* and *RNF170* caused similar clinical phenotypes, as both resulted in increased IP3R1 protein levels and ER calcium release. Previous studies have found that ERLIN2 recruits RNF170 to ubiquitinate activated IP3R1 and degrades it through the ubiquitin protease pathway [29]. In addition, RNF170 is also involved in the turnover of IP3R1 at the basal level. Therefore, RNF170 knockdown leads to increased IP3R1 expression. In the present study, we found that the p.Val71Ala heterozygous missense variant of *ERLIN2* gained function by recruiting a novel E3 ubiquitin ligase RNF213, resulting in the degradation of IP3R1 and decreased expression levels. Contrarily, the gain-of-function variants in *ERLIN2* and *RNF170* would result in the degradation of IP3R1, which blocked the release of calcium from the ER. Interestingly, heterozygous deletion or biallelic variant in *IP3R1* has been found to be related to SCA15 (childhood to adulthood onset) and SCA29 (neonatal onset) separately [21, 30], which mimic the clinical features of HSP. These imply that its loss of function is linked to motor neuron developmental disorders. Thus, both gain-of-function and loss-of-function variants in these genes are made for motor neuron disorders by disrupting intracellular calcium homeostasis.

Previous studies on HSP concentrated on the degenerative alterations in motor neurons. The *RNF170* homozygous knockout mice developed sensory and gait abnormalities in old age [31], suggesting that HSP was a neurodegenerative disease. However, some researchers believed that the pathogenesis mechanisms of HSP might also involve the disturbance of neurodevelopment. In the *RNF170* knockdown zebrafish model, Wagner et al. observed neurodevelopment defects at 48 hpf, such as a shortened body axis, microphthalmia, microcephaly, and alterations in pigmentation [32]. Therefore, as illustrated in other neurodegenerative disorders, such as AD [33] and PD [34], HSP might also manifest neurodevelopment or neurodifferentiation perturbations in the early stage. In the present study, we explored the relevant mechanisms from a neurodevelopmental perspective. Using the human iPSC-based models, we discovered that ERLIN2 with p.Val71Ala degraded IP3R1 by recruiting the E3 ubiquitin ligase RNF213. The decrease of IP3R1 blocked calcium efflux in the ER, which led to ER stress-mediated apoptosis. Meanwhile, disturbance of intracellular calcium homeostasis affected multiple signaling pathways, resulting in decreased NSC proliferation and blocked motor neuron differentiation. In addition, different HSP genes such as SPG4 and SPG11 affect cell proliferation, cell cycle, and neurite development during neurogenesis [35].

In summary, we explored the mechanism of the *ERLIN2* p.Val71Ala variant in neural development based on the stem cell model. We discovered that the variant of *ERLIN2* acquires a novel function by recruiting the E3 ubiquitin ligase RNF213, which degrades IP3R1 to disrupt intracellular calcium homeostasis. However, *in vivo* models are needed to evaluate the phenotype and mechanism of *ERLIN2* deletion and mutant knock-in. The mechanism of other heterozygous variant sites needs to be investigated properly, to guide our subsequent research.

## Figures and Tables

**Table 1:** The clinical features of the member in the *ERLIN2* p.Val71Ala-mutant family.

Patients	II-2	II-5	II-3	III-1
Age at onset (y)	32	13	54	28
Sex	F	M	F	F
Sensation	NA	NA	NA	NA
Muscular tension	Increase	Increase	Increase	NA
Myodynamia	3 grade	4 grade	5 grade	5 grade
Abnormal gait	Yes	Yes	Yes	No
Fasciculation	Yes	Yes	Yes	No
Spasticity	Yes	Yes	Yes	No
Pathological condition	Yes	Yes	ND	NA
Tendon reflex	Hyperreflexia	Hyperreflexia	Hyperreflexia	NA
Ataxia	Yes	ND	NA	NA
Lalopathy	Yes	ND	NA	NA
Visual impairment	Yes	ND	NA	NA
Cognitive disorder	Yes	ND	NA	NA
SPRS score	44	10	39	8
PROVEAN score			-3.255 (deleterious)	
FATHMM score			-3.64 (damaging)	
MutationTaster rank score			1 (disease causing)	

F: female; M: male; NA: not abnormal; ND: no data.

**Table 2:** The clinical features of the families containing heterozygosis missense variants in *ERLIN2* that cause HSP.

Family	I	II	III	IV	V
Age at onset (y)	15-38	9-46	32	20-25	25-45
Variant	c.452 C>T, p.Ala151Val	c.386G>C, p.Ser129Thr	c.187C>A, p.Gln63Lys	c.206A>T, p.D69V	c.502G>A, p.V168M
Sensation	Yes	ND	No	No	No
Muscular tension	LL	LL	NA	LL	LL
Myodynamia	LL 2-4	LL 1-4	LL	LL 1-4	LL
Abnormal gait	Yes	Yes	Yes	Yes	Yes
Fasciculation	Yes	Yes	No	Yes	Yes
Spasticity	LL	LL	LL	LL	LL
Pathological condition	Yes	Yes	No	Yes	Yes
Tendon reflex	LL	LL	NA	LL	LL
Ataxia	Yes	Yes	Yes	Yes	Yes
Lalopathy	No	No	No	Yes	No
Visual impairment	No	No	No	No	No
Intellectual disability	No	No	No	No	No
Skeletal deformity	Yes	No	No	No	No

LL: lower limbs; NA: not abnormal; ND: no data.

**Table 3:** Clinical features of lipid raft disease caused by different genes.

Genes	Gene-encoded proteins	Inheritance	Phenotype	Clinical features
*RNF170*	Ring finger protein 170 (RNF170)	AD	Sensory ataxia type 1 (SNAX1)	Ear vestibular areflexia Gait ataxia, hyporeflexia, areflexia Distal sensory loss to all modalities (lower limbs more affected than upper limbs) Adult onset (range 28 to 55 years) Slowly progressive
		AR	Hereditary spastic paraplegia type 85 (SPG85)	Optic atrophy Dysphagia, delayed motor development, spastic paraplegia, hyperreflexia of the lower limbs, ataxic gait, loss of ambulation, dysarthria, upper limb involvement-later onset, cerebellar atrophy Axonal polyneuropathy, later onset Onset in the first years of life

*ERLIN2*	Endoplasmic reticulum lipid raft-associated protein 2 (ERLIN2)	AD	Hereditary spastic paraplegia	Muscle weakness, muscle atrophy, increased muscle tone, abnormal gait, lower limb spasticity, upper limb spasticity-mild, hyperreflexia, lack of speech Intellectual disability Dysphagia Later onset
		AR	Hereditary spastic paraplegia type 18 (SPG18)	Eye squint Spine scoliosis and kyphosis, muscle weakness, muscle atrophy, increased muscle tone, abnormal gait, lower limb spasticity, upper limb spasticity-mild, extensor plantar responses, hyperreflexia, lack of speech, intellectual disability, seizures Onset in infancy or childhood (range 1 to 6 years) Regression in infancy Results in severe motor disability and loss of independent ambulation

*ERLIN1*	Endoplasmic reticulum lipid raft-associated protein 1 (ERLIN1)	AR	Hereditary spastic paraplegia type 62 (SPG62)	Spine thoracic scoliosis, flexion contractures of the knees, amyotrophy, lower limb spasticity, spastic gait, walking on tiptoes, hyperreflexia, clonus, absent patellar reflexes Absent Achilles tendon reflexes Dysarthria Age at onset, 20 months to 13 years
		AD	?	

*IPRR1*	Inositol 1,4,5-triphosphate receptor, type 1 (IP3R1)	AD	Spinocerebellar ataxia type 15 (SCA15)	Eye dysmetric saccades, nystagmus, horizontal, gaze-evoked Systemic ataxia, dysarthria, scanning speech, hyperreflexia, action tremor, postural tremor, hyperreflexia, cerebellar atrophy Wide range of onset from childhood to adult (10 to 50 years) Very slow progression Most patients remain ambulatory
		AD	Spinocerebellar ataxia type 29 (SCA29)	Eyes: nystagmus and saccadic eye movements Cerebellar ataxia, nonprogressive, delayed motor development, broad-based gait, limb ataxia, dysarthria, dysdiadochokinesis, intention tremor, dysmetria, nystagmus Cognitive impairment-mild Atrophy of the cerebellar vermis seen on MRI Onset at birth
		AR/AD	Gillespie syndrome (GLSP)	Eyes: iris hypoplasia Scalloped pupillary margins of iris, nystagmus, visual impairment, mild to moderate, general hypotonia, delayed motor development, ataxia, postural tremor, slurred speech Intellectual disability-mild to severe Cerebellar hypoplasia/atrophy

AR: autosomal recessive; AD: autosomal dominant.

## Data Availability

The data of the findings in this study are available from the corresponding authors upon reasonable request.

## Supplementary Materials

Supplementary Materials The supplementary material contains Supplementary Figures 1–4 and Supplementary Tables S1 and S2; the figure legends and table legends are available in the supplementary files.
