# Supplementary material for: Disruption of Intracellular Calcium Homeostasis Leads to ERLIN2-Linked Hereditary Spastic Paraplegia in Patient-Derived Stem Cell Models
Source: Hum Mutat. 2023 Jun 16;2023:4834423. doi: 10.1155/2023/4834423 (PMC11919107; doi:10.1155/2023/4834423)
Supplement: Supplementary Materials — The supplementary material contains Supplementary Figures 1–4 and Supplementary Tables S1 and S2; the figure legends and table legends are available in the supplementary files. [file 4834423.f1.zip › revised Supplemental Figures.docx]

**Supplemental Figures**

**Disruption of intracellular calcium homeostasis leads to ERLIN2-linked hereditary spastic paraplegia in patient-derived stem cell models**

**Xintong Zhu^1*^, Xiaoyin Tan^1,2*^,** **Junwen Wang^1^, Limeng Dai^1^,** **Jia Li^1^, Xingying Guan^1^,** **Ziyi Wang^1^, Mao Zhang^1^, Jun Hu^3^,** **Yun Bai^1,4#^, Hong Guo^1,4#^**

**Correspondence:** Hong Guo: guohong02@gmail.com; Yun Bai: [yunbai@tmmu.edu.cn](mailto:yunbai@tmmu.edu.cn)

**Supplemental Figures**

**
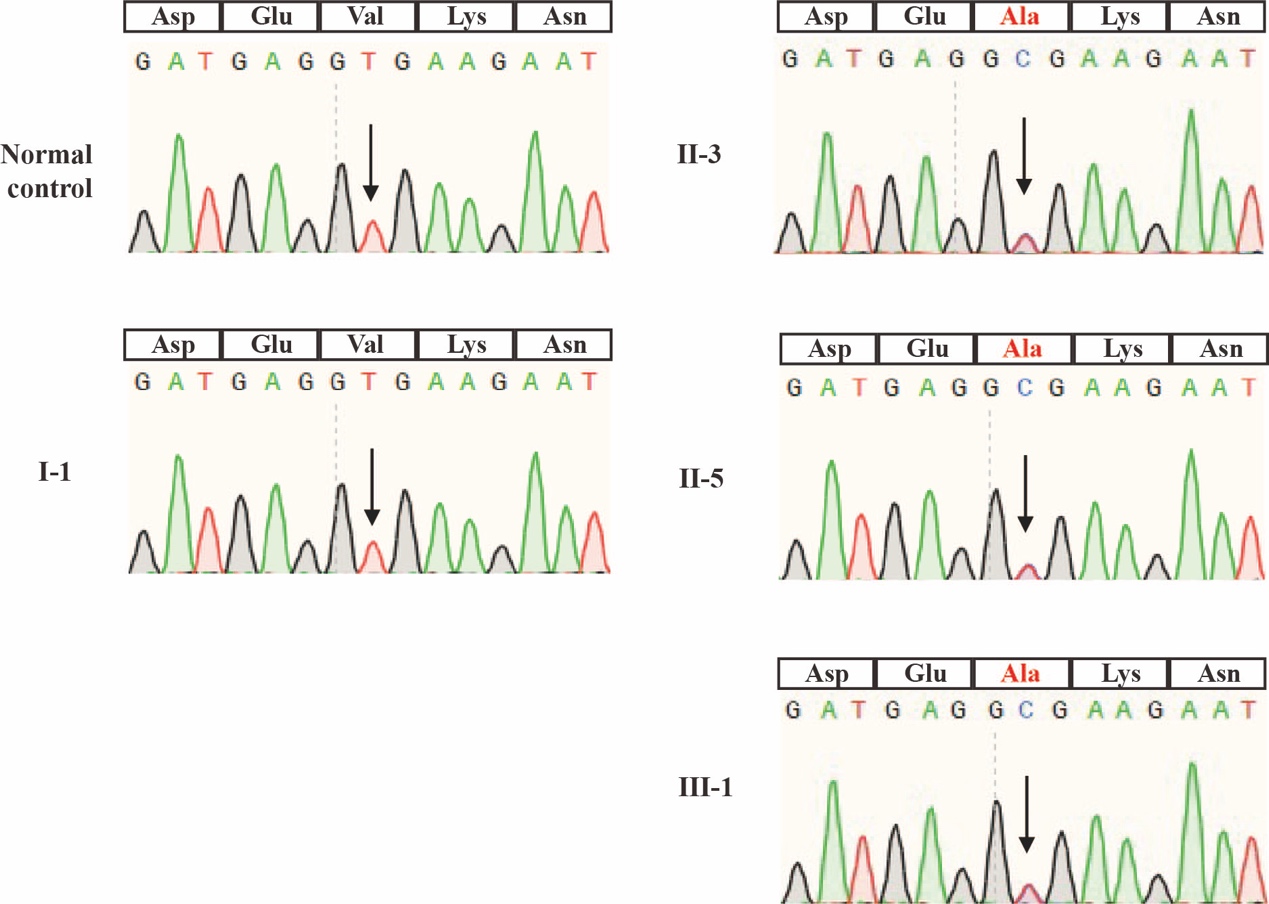
**

**Supplemental Figure 1.** The partial nucleotide sequences of exon 4 of *ERLIN2* show the normal sequence in the unaffected individuals (I-1) and the c.212 T > C mutation in the affected family members (II-2, II-3, II-5, III-1).

**
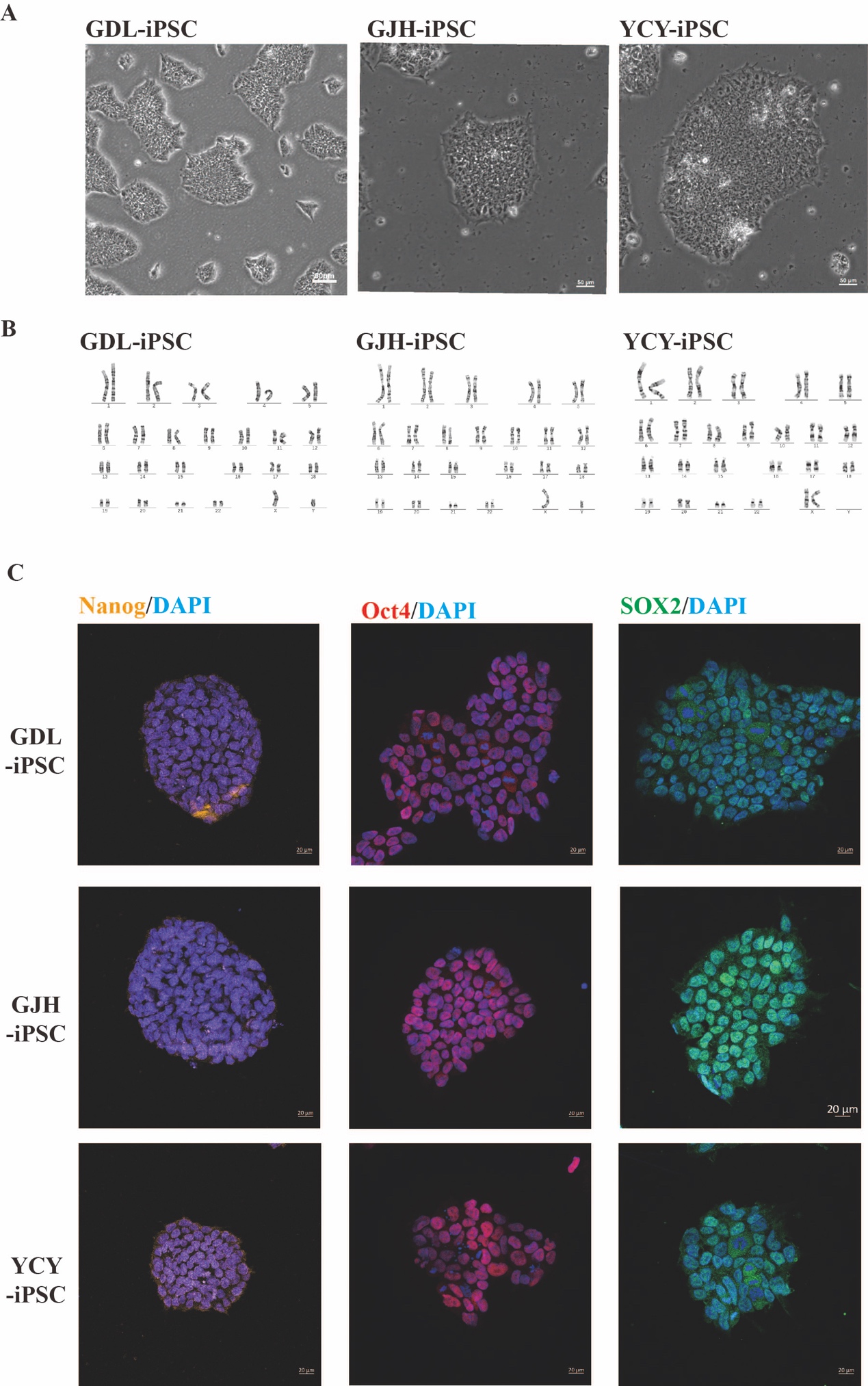
**

**Supplemental Figure 2.** (A) Microscopic observation of clonal clusters in three iPSC lines. (B) Karyotype analysis of three iPSC lines showed no numerical or structural abnormalities of chromosomes. (C) Pluripotency was detected by immunofluorescence, Nanog (yellow), OCT4 (red), SOX2 (green), and nuclear raiser (blue).

**
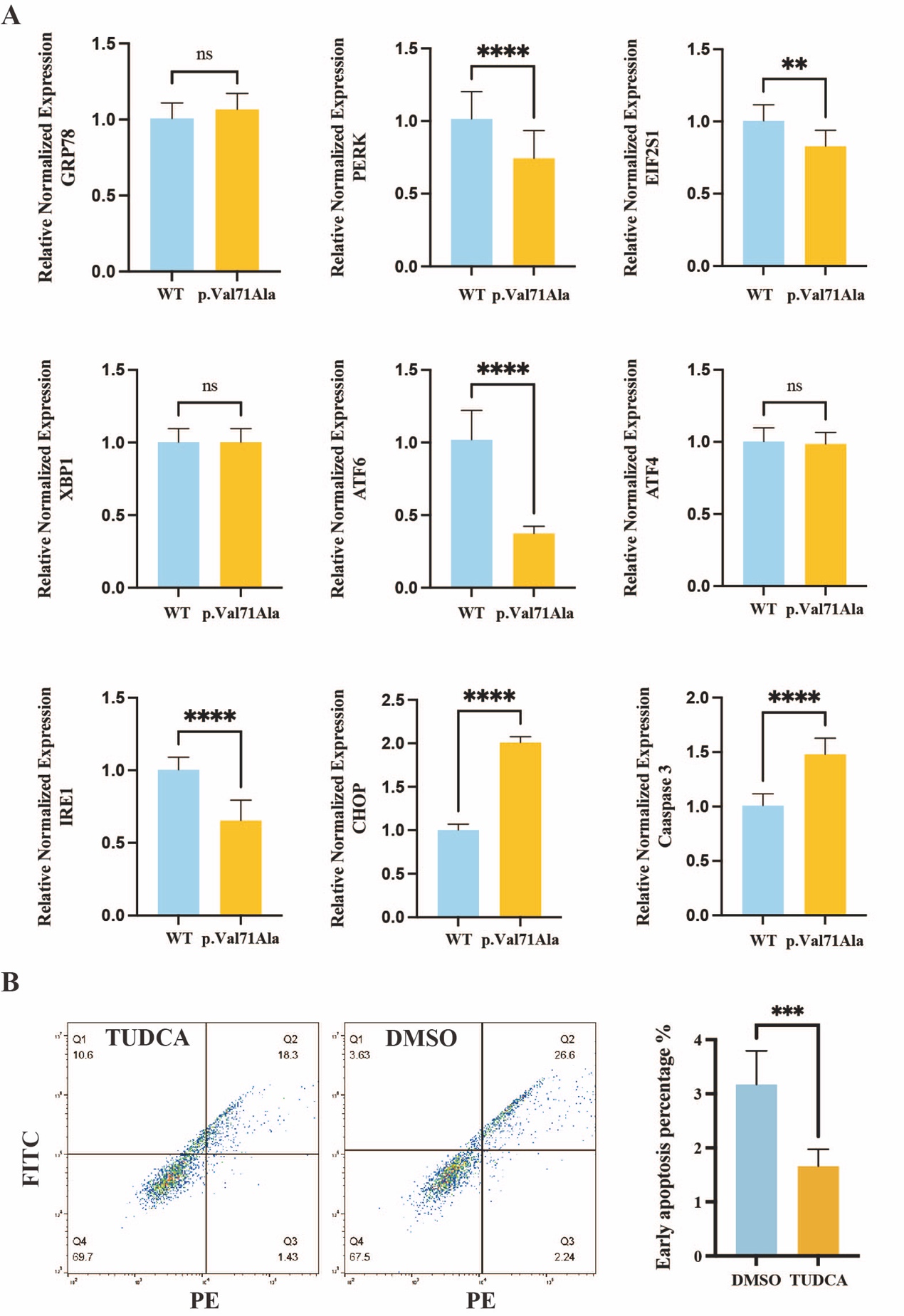
**

**Supplemental Figure 3.** (A) In the RT-PCR of genes that related to UPR, the mRNA levels of GRP78, XBP1, and ATF4 were not significant difference, while the mRNA levels of PERK, EIF2S1, ATF6, and IRE1 were significantly decreased in p.Val71Ala group, the mRNA levels of CHOP and Caspase 3 were prominent increased (*mean ± SD*, *N*=3 experiments, **p*<0.05, ***p*0.01, *****p*<0.0001). (B) The WT was treated with 100 uM DMSO and 100uM tauroursodeoxycholate (TUDCA), Apoptosis was detected by flow cytometry after staining with Annexin V-FITC Apoptosis Detection Kit, and the early apoptosis percentage was expressed as Q3. The bar chart represents the quantification of the result (*mean ± SD*, *N*=3 experiments, *****p*<0.0001).


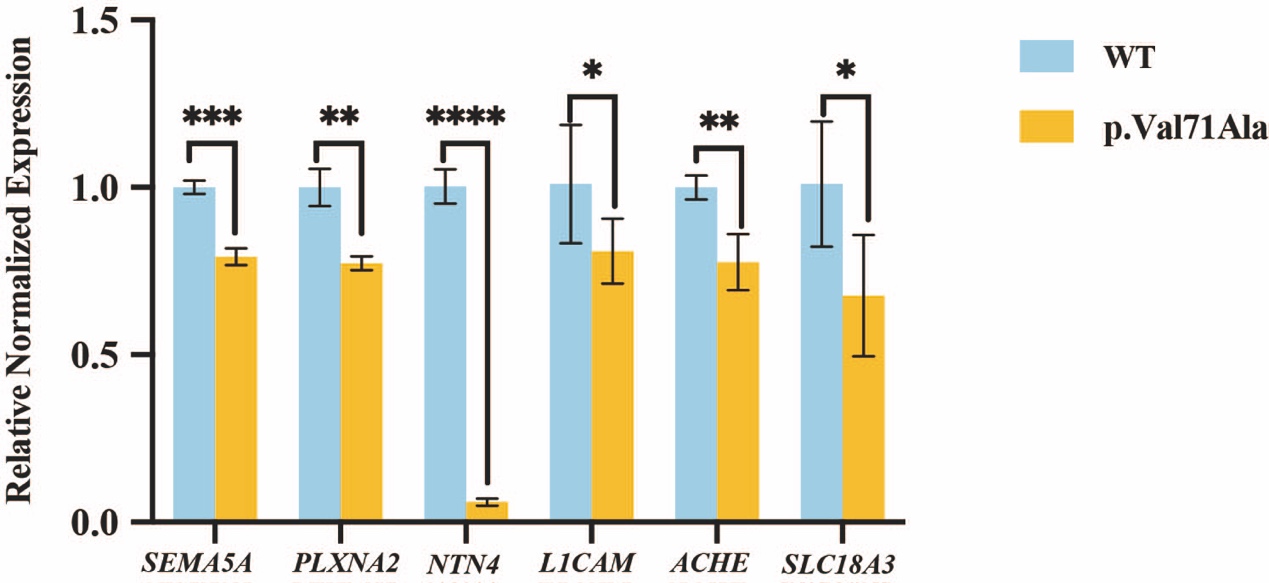


**Supplemental Figure 4.** The RT-PCR for genes that promote motor neuron differentiation (*mean ± SD*, *N*=3 experiments, ***p*<0.01, *****p*<0.0001, *^ns^p*>0.05).
