# Supplementary material for: Disruption of Intracellular Calcium Homeostasis Leads to ERLIN2-Linked Hereditary Spastic Paraplegia in Patient-Derived Stem Cell Models
Source: Hum Mutat. 2023 Jun 16;2023:4834423. doi: 10.1155/2023/4834423 (PMC11919107; doi:10.1155/2023/4834423)
Supplement: Supplementary Materials — The supplementary material contains Supplementary Figures 1–4 and Supplementary Tables S1 and S2; the figure legends and table legends are available in the supplementary files. [file 4834423.f1.zip › revised Supplemental Table.docx]

**Supplemental Tables**

**Disruption of intracellular calcium homeostasis leads to ERLIN2-linked hereditary spastic paraplegia in patient-derived stem cell models**

**Xintong Zhu^1*^, Xiaoyin Tan^1,2*^,** **Junwen Wang^1^, Limeng Dai^1^,** **Jia Li^1^, Xingying Guan^1^,** **Ziyi Wang^1^, Mao Zhang^1^, Jun Hu^3^,** **Yun Bai^1,4#^, Hong Guo^1,4#^**

**Correspondence:** Hong Guo: guohong02@gmail.com; Yun Bai: [yunbai@tmmu.edu.cn](mailto:yunbai@tmmu.edu.cn)

**Supplemental Table S1. Spastic Paraplegia Rating Scale**

| **Item** | **Scores** | | | |
| --- | --- | --- | --- | --- |
|  | **II-2** | **II-3** | **II-5** | **III-1** |
| **1.Walking distance without pause** | 4 | 1 | 4 | 0 |
| **2.Gait quality** | 4 | 1 | 4 | 1 |
| **3.Maximum gait speed** | 4 | 1 | 4 | 1 |
| **4.Climbing stairs** | 4 | 1 | 3 | 1 |
| **5.Speed of stairs climbing** | 4 | 1 | 3 | 1 |
| **6.Arising from chair** | 4 | 0 | 3 | 0 |
| **7.Spasticity, hip addutor muscles** | 3 | 1 | 4 | 1 |
| **8.Spasticity, keen extension** | 4 | 1 | 4 | 1 |
| **9.Weakness, hip abduction** | 4 | 1 | 3 | 1 |
| **10.Weakeness, foot dorsiflexion** | 4 | 1 | 3 | 1 |
| **11.Cobtractures of lower limbs** | 2 | 1 | 1 | 0 |
| **12.Pain due to SP-related symptoms** | 2 | 0 | 2 | 0 |
| **13.Bladder and bowel function** | 1 | 0 | 1 | 0 |
| **Total** | 44 | 10 | 39 | 8 |

SP: spastic paralegia

**Supplemental Table S2. The details of qRT–PCR primers**

| **Primers** | **Sequences** |
| --- | --- |
| *CASP 3* | F: 5'-CATGGAAGCGAATCAATGGACT-3';  R: 5'-CTGTACCAGACCGAGATGTCA-3' |
| *GRP78*  *(HSPA5)* | F: 5'-GAAAGAAGGTTACCCATGCAGT-3';  R: 5'-CAGGCCATAAGCAATAGCAGC-3' |
| *CHOP*  *(DDIT3)* | F: 5'-GAACGGCTCAAGCAGGAAATC-3';  R: 5'-TTCACCATTCGGTCAATCAGAG-3' |
| *IRE1*  *(ERN1)* | F: 5'-CACAGTGACGCTTCCTGAAAC-3';  R: 5'-GCCATCATTAGGATCTGGGAGA-3' |
| *PERK*  *(EIF2AK3)* | F: 5'-ACGATGAGACAGAGTTGCGAC-3';  R: 5'-ATCCAAGGCAGCAATTCTCCC-3' |
| *ATF6* | F: 5'-TCCTCGGTCAGTGGACTCTTA-3'; ' |
|  | R: 5'-CTTGGGCTGAATTGAAGGTTTTG-3 |
| *XBP1* | F: 5'-CCCTCCAGAACATCTCCCCAT-3';  R: 5'-ACATGACTGGGTCCAAGTTGT-3' |
| *ATF4* | F: 5'-CCCTTCACCTTCTTACAACCTC-3';  R: 5'-TGCCCAGCTCTAAACTAAAGGA-3' |
| *EIF2S1* | F: 5'-TGGTGAATGTCAGATCCATTGC-3';  R: 5'-TAGAACGGATACGCCTTCTGG-3' |
| *SLC18A3* | F: 5'-TTCGCCTCTACAGTCCTGTTC-3';  R: 5'-GCTCCTCCGGGTACTTATCG-3' |
| *ACHE* | F: 5'-GGGTGGTAGACGCTACAACC-3';  R: 5'-GTGCCCTCAAAACCTGGGTAT-3' |
| *NTN4* | F: 5'-CCTGCGGTCAGAATGCTACC-3';  R: 5'-GAGGAAACCGGAAGGATGAGT-3' |
| *SEMA5A* | F: 5'-GGAACCTGTGTTATAGCATGGC-3';  R: 5'-GCACTGAGTCGTACCCTGG-3' |
| *PLXNA2* | F: 5'-CTACCTGTCCAGTGTCAACAAG-3';  R: 5'-GGTCGGGAAGTAATCCTGCTT-3' |
| *L1CAM* | F: 5'-CCGACAACCACTCAGACTACA-3';  R: 5'-CCGGAGGTCAATGGGTTCC-3' |

F, Forward Primer；R，Reverse Primer.
